# Clinical and virological findings in patients with Usutu virus infection, northern Italy, 2018

**DOI:** 10.2807/1560-7917.ES.2019.24.47.1900180

**Published:** 2019-11-21

**Authors:** Monia Pacenti, Alessandro Sinigaglia, Thomas Martello, Elena De Rui, Elisa Franchin, Silvana Pagni, Elektra Peta, Silvia Riccetti, Adelaide Milani, Fabrizio Montarsi, Gioia Capelli, Carlo Giovanni Doroldi, Francesco Bigolin, Luca Santelli, Lucia Nardetto, Marco Zoccarato, Luisa Barzon

**Affiliations:** 1 Microbiology and Virology Unit, Padua University Hospital, Padova, Italy; 2 These authors contributed equally as first authors; 3 Department of Molecular Medicine, University of Padova, Padova, Italy; 4 Istituto Zooprofilattico Sperimentale delle Venezie, Legnaro PD, Italy; 5 Medicine Unit, Camposampiero Hospital, Azienda ULSS 6 Euganea, Padova, Italy; 6 Neurology Department, Ospedale S. Antonio, Azienda ULSS 6 Euganea, Padova, Italy

**Keywords:** Usutu virus, West Nile Virus, encephalitis, fever, Italy, human infection

## Abstract

**Background:**

Usutu virus (USUV) is a mosquito-borne flavivirus, which shares its transmission cycle with the phylogenetically related West Nile virus (WNV). USUV circulates in several European countries and its activity has increased over the last 5 years.

**Aim:**

To describe human cases of USUV infection identified by surveillance for WNV and USUV infection in the Veneto Region of northern Italy in 2018.

**Methods:**

From 1 June to 30 November 2018, all cases of suspected autochthonous arbovirus infection and blood donors who had a reactive WNV nucleic acid test were investigated for both WNV and USUV infection by in-house molecular methods. Anti-WNV and anti-USUV IgM and IgG antibodies were detected by ELISA and in-house immunofluorescence assay, respectively; positive serum samples were further tested by WNV and USUV neutralisation assays run in parallel.

**Results:**

Eight cases of USUV infection (one with neuroinvasive disease, six with fever and one viraemic blood donor who developed arthralgia and myalgia) and 427 cases of WNV infection were identified. A remarkable finding of this study was the persistence of USUV RNA in the blood and urine of three patients during follow-up. USUV genome sequences from two patients shared over 99% nt identity with USUV sequences detected in mosquito pools from the same area and clustered within lineage Europe 2.

**Conclusions:**

Clinical presentation and laboratory findings in patients with USUV infection were similar to those found in patients with WNV infection. Cross-reactivity of serology and molecular tests challenged the differential diagnosis.

## Introduction

Usutu virus (USUV) is a mosquito-borne member of the *Flavivirus* genus, family *Flaviviridae*, classified in the Japanese encephalitis antigenic complex together with the closely phylogenetically related West Nile virus (WNV). The enzootic transmission cycles and geographical distribution of USUV and WNV often overlap, as both viruses are transmitted by ornithophilic mosquito species, mainly *Culex* spp., and amplified by a variety of migratory and resident bird species [1]. Humans and other mammals may be incidentally infected, but their low-level viraemia does not allow further transmission through a mosquito bite.

WNV is a well-recognised human pathogen, which causes neuroinvasive disease (i.e. encephalitis, meningitis or acute flaccid paralysis) in ca 1/150 infected individuals and influenza-like illness (ILI) in ca 20–30% of infections. Risk groups for WNV infection are elderly people above 65 years of age and immunocompromised patients. USUV appears to be more pathogenic and fatal for some bird species than WNV, but it rarely causes disease in humans. USUV was first isolated from mosquitoes in South Africa in 1959 [2] and first identified in Europe in 2001, where it was responsible for several deaths in various bird species in Austria [3]. A retrospective analysis carried out by Weissenböck et al. found USUV in archived tissue samples from blackbirds from 1996 in the Tuscany region of Italy, which could suggest that the virus had been introduced into Europe earlier than 2001 [4]. USUV circulates in several countries in central and western Europe and its activity has increased in the last 5 years, especially in Germany, France, Belgium and the Netherlands [1].

Seroprevalence studies in Italy indicate that the prevalence of antibodies against USUV in humans is higher than anti-WNV antibodies in areas where both viruses co-circulate [5-8], supporting the speculation that most human USUV infections are asymptomatic. Several cases of asymptomatic USUV infection were accidently identified when blood donors were screened with WNV nucleic acid amplification tests (NAT) that cross-react with other flaviviruses [9-14]. Symptomatic USUV infections in humans are uncommon; only 20 cases have been described in the literature so far including: (i) two cases with fever from Africa [15], (ii) one blood donor with a rash from Austria [14], (iii) 13 cases with neuroinvasive disease from Italy [6,16,17], (iv) three cases presenting as meningoencephalitis, encephalitis and polyneuritis from Croatia [18], and (v) one case with facial paralysis from France [19].

In Italy, USUV infection is a notifiable disease and USUV surveillance has been included in the national plan since 2017 [20].

To improve knowledge on USUV disease in humans, we describe clinical and virological findings and the results of follow-up investigation of eight symptomatic USUV infection cases identified in the Veneto Region of northern Italy during the 2018 transmission season.

## Methods

The Italian Ministry of Health publishes an annually revised surveillance plan, which aims to reduce the risk of WNV transmission to humans by detecting viral circulation early and triggering both vector-control measures and substances of human origin safety measures [20]. Based on this plan, all suspected autochthonous arboviral infections are tested at the regional reference laboratory of Veneto Region for confirmation of WNV and USUV infection.

### Study design and data collection

During the surveillance period (1 June–30 November 2018), a total of 1,967 cases of suspected autochthonous arboviral infection were tested at the regional reference laboratory of Veneto Region (Microbiology and Virology Unit, Padova University Hospital, Italy) for confirmation of WNV and USUV infection. According to the surveillance plan, information about clinical symptoms, vaccinations against flaviviruses, previous infections, and recent travels history were collected in case report forms. For the USUV cases described in this study, further clinical information, laboratory data, and imaging results were extracted from the medical records during hospitalization. According to the national plan, entomological surveillance was activated from May to October in the Veneto Region, using 55 Centre for Disease Control and Prevention (CDC) light traps baited with carbon dioxide capturing mosquitoes for 1 night every 15 days.

Case definition of WNV and USUV infection was according to the national surveillance plan [20] (Box).

Box **Case definition of West Nile virus and Usutu virus infection, Veneto region, northern Italy, 2018**

**Confirmed case:** individuals presenting with at least one of the following laboratory criteria:

• virus isolation from serum, urine, and/or CSF;

• detection of viral RNA in blood, urine, and/or CSF;

• detection of a specific IgM antibody response in CSF;

• high IgM antibody titre and detection of IgG antibodies in serum and confirmation by neutralisation assays.

**Probable case:** Individuals with only IgM antibodies detected in serum.

CSF: cerebrospinal fluid.

### Laboratory methods

During the surveillance period (1 June–30 November 2018), all cases of suspected autochthonous arboviral infection and all blood, tissue and organ donors who had a reactive WNV NAT were tested for both WNV and USUV infection by in-house real-time PCR methods. Specifically, for USUV and WNV RNA detection, total nucleic acids were purified from 200 μL whole blood by using a MagNA Pure 96 System (Roche Applied Sciences, Basel, Switzerland) and from 1,000 μL plasma, urine or cerebrospinal fluid (CSF) by using a NucliSens EasyMag System (BioMerieux, Marcy-l'Étoile, France). Detection of WNV RNA was performed by in-house real-time RT-PCR methods, as previously described [21]. Detection of USUV RNA was performed by an in-house real-time RT-PCR assay targeting the NS5 gene [22]; positive samples were tested for confirmation with a second in-house real-time RT-PCR assay targeting the NS1 gene [23]. Real-time RT-PCR assays were carried out using AgPath-ID™ One-Step RT-PCR Reagents (Thermo Fisher Scientific, Waltham, Massachusetts, United States (US)) and run on ABI 7900HT Sequence Detection Systems (Thermo Fisher Scientific).

USUV genotyping and phylogenetic analysis were based on sequencing partial regions of the E, NS3, and NS5 genes, as reported in [24] and by pan-flavivirus nested RT-PCR and sequencing [25]. Sequencing was performed using a Big Dye 3.1 kit and run on an ABI PRISM 3130xl Genetic Analyzer (Thermo Fisher Scientific).

The presence of WNV IgM and IgG antibodies in serum and CSF was determined by a commercial ELISA (WNV IgM capture DxSelect and IgG DxSelect, Focus Diagnostics, California, US). The presence of USUV IgM and IgG antibodies in serum was determined by an immunofluorescence assay (IFA) developed in-house. Briefly, the IFA assay was developed by seeding Vero cells infected with USUV and (to control for specificity) WNV at MOI 0.01 on microscope glass slides at a density of 2,000 cells/well. After fixing the cells on the slide, 20 µL of serum samples were added to the wells in duplicate. The slides were then incubated at 37 °C for 1 hour, followed by staining with a FITC-labelled anti-human IgG antibody. Serum samples with positive WNV ELISA and/or USUV IFA results were further tested for confirmation by WNV and USUV neutralisation assays run in parallel. Detection of WNV and USUV neutralising antibodies was performed by plaque reduction neutralisation test (PRNT) and microneutralisation titre assay (MNTA), respectively, on Vero cells. The titres of WNV- and USUV-neutralising antibodies were defined, respectively, as the reciprocal of the highest dilution of the serum that reduced by 50% the number of plaques in Vero cells (PRNT50) and as the reciprocal of the highest dilution of the serum that showed 100% neutralisation of cytopathic effect in MNTA. Neutralising antibody titres ≥ 20 were considered positive.

Mosquitoes were morphologically identified, pooled (100 specimens maximum) and screened for flaviviruses by using a one-step SYBR green-based real-time RT-PCR targeting 250 bp of the conserved region of the non-structural NS5 gene as described elsewhere [26]. All Flavivirus-positive samples were directly sequenced to differentiate WNV, USUV or other flaviviruses.

### Ethical statement

Since the cases reported in this study were investigated with routine procedures according to the national surveillance plan for WNV and USUV infection, no approval was required from the ethics committee. Written informed consent was obtained from the patients described in this report.

## Results

### Human and entomological surveillance of USUV and WNV infection

During the surveillance period (1 June–30 November 2018), the regional reference laboratory identified eight human cases of USUV infection (one with neuroinvasive disease, six with fever and one viraemic blood donor) and 427 human cases of WNV infection (86 with neuroinvasive disease, 307 with fever, and 34 viraemic blood donors). Among 34 blood donors with positive WNV NAT, 26 had a confirmed diagnosis of WNV infection by detection of WNV RNA in blood and/or urine by in-house real-time PCR, viral genome sequencing and appearance of WNV IgM and IgG antibodies. In the remaining eight blood donors, WNV infection was confirmed by testing WNV and USUV neutralising antibodies in parallel, while WNV RNA and USUV RNA were undetectable by in-house methods.

During the same period, entomological surveillance identified 85 *Culex* spp. mosquito pools positive for USUV and 155 *Culex* spp. mosquito pools positive for WNV of 1,247 tested pools.

The total number of West Nile cases identified in 2018 was ca 10-fold higher than the average number of cases reported during the previous 5 years in the Veneto Region. No human cases of USUV infection were notified in the previous years. The number of WNV- and USUV-positive mosquito pools was also higher than in the previous years, with about sevenfold increase for WNV and threefold increase for USUV.

### Description of human cases of USUV infection

In three of eight cases of USUV infection, diagnosis was based on detection of USUV RNA in body fluids and demonstration of a specific antibody response, which was confirmed by neutralisation assay. In the other five cases, diagnosis was confirmed by demonstration of seroconversion and positive neutralisation assay (Table 1). One of these patients had symptoms of encephalitis, six had ILI and the eighth, who was identified by screening of blood donors with WNV NAT, developed mild symptoms.

**Table 1:** Clinical and laboratory findings patients with Usutu virus infection, northern Italy, 2018 (n = 8)

Parameter	Total/cases with available information
Sex	7 M/1 F
Median age in years (range)	64 (17–91)
Median days since symptom onset or index blood donation median (range)	7 (2–24)
**Symptoms**	
Fever	7/8
Arthralgia	2/8
Myalgia	5/8
Headache	4/8
Asthenia	5/8
Rash	2/8
Meningitis	0/8
Encephalitis	1/8
Acute flaccid paralysis	0/8
**Laboratory findings**	
USUV RNA in blood	3/4
USUV RNA in urine	3/4
USUV RNA in CSF	0/1
USUV RNA in saliva	0/1
USUV IgM positive (in-house IFA)	8/8
USUV IgG positive (in-house IFA)	6/8
WNV RNA in blood	0/4
WNV RNA in urine	0/4
WNV IgM positive (ELISA)	8/8
WNV IgG positive (ELISA)	4/8
USUV MNTA positive	8/8
WNV PRNT positive	7/8
USUV NT > WNV NT^a^	8/8

CSF: cerebrospinal fluid; ELISA: enzyme-linked immunosorbent assay; F: female; IFA: immunofluorescence assay; M: male; MNTA: microneutralisation titre assay; NT: neutralising antibody titre; PRNT: plaque reduction neutralisation test; USUV: Usutu virus; WNV: West Nile virus.

^a^ In all patients, USUV NT were at least fourfold higher than WNV NT.

**Figure 1:**
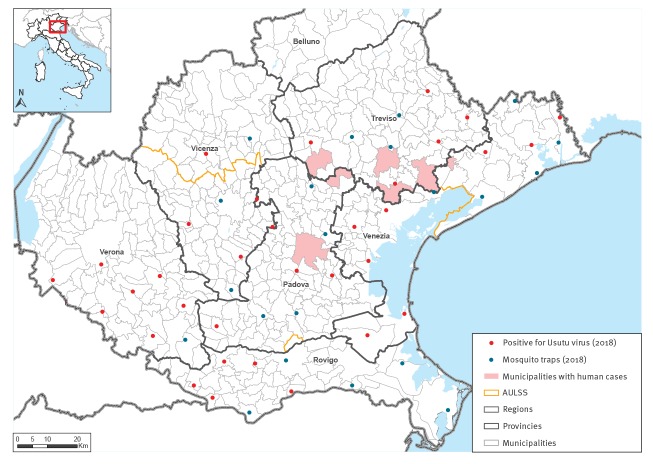
Geographical distribution of USUV-positive *Culex* mosquito pools and human cases of USUV infection, Veneto Region, northern Italy, 2018

Most Usutu cases lived near the borders of Padua, Treviso and Venice provinces (Figure 1). Cases were classified as autochthonous since the patients did not travel abroad during the 28 days before symptoms onset. Symptom onset occurred between mid-July and mid-September 2018, corresponding to the period of USUV activity in the Veneto Region. The main clinical and laboratory features of USUV cases are summarised in Tables 1 and 2 and described in detail below.

USUV: Usutu virus.

Sites of mosquito traps are represented with dots. Red dots indicate sites where USUV-positive mosquito pools were collected, blue dots indicate sites where no USUV-positive mosquito pools were detected. Pink areas represent municipalities where human cases of USUV infection were present.

Source: Istituto Zooprofilattico delle Venezie (IZSVe).

**Table 2:** Follow-up evaluation of three cases of USUV infection with detectable USUV RNA in blood, northern Italy, 2018

Characteristics	Case 1					Case 2			Case 3	
	Aphasia, apraxia, temporal disorientation					Fever, headache, arthralgia			Fever, headache, myalgia	
Days since symptom onset	3	8	9	10	40	24	28	34	15	23
USUV RNA in blood^a^	Pos (34.7)	Pos (36.1)	Pos (35.86)	Pos (31.8)	Pos (33.5)	Pos (28.4)	Pos (28.1)	Pos (28.7)	Pos (28.3)	Pos (30.2)
USUV RNA in urine^a^	ND	Pos (34.1)	Neg	Neg	Neg	Pos (29.1)	Pos (30.8)	Neg	Pos (28.9)	Pos (30.0)
USUV RNA in CSF	Neg	ND	ND	ND	ND	ND	ND	ND	ND	ND
USUV RNA in saliva	ND	ND	ND	ND	ND	ND	Neg	ND	ND	ND
USUV IgM IFA	Pos	ND	ND	ND	Pos	Pos	ND	Pos	Pos	Pos
USUV IgG IFA	Neg	ND	ND	ND	Pos	Pos	ND	Pos	Pos	Pos
USUV NT	80	ND	ND	ND	160	160	ND	320	320	480
WNV NAT in plasma	Pos	ND	ND	ND	ND	ND	ND	ND	ND	ND
WNV RNA in blood	Neg	Neg	Neg	Neg	Neg	Neg	Neg	Neg	Neg	Neg
WNV RNA in urine	Neg	Neg	Neg	Neg	Neg	Neg	Neg	Neg	Neg	Neg
WNV RNA in CSF	Neg	ND	ND	ND	ND	ND	ND	ND	ND	ND
WNV RNA in saliva	ND	ND	ND	ND	ND	ND	Neg	ND	ND	ND
WNV IgM ELISA	Pos	ND	ND	ND	Pos	Pos	ND	Pos	Pos	Pos
WNV IgG ELISA	Neg	ND	ND	ND	Neg	Neg	ND	Neg	Neg	Pos
WNV NT	< 20	ND	ND	ND	< 20	< 20	ND	20	20	40
Flavivirus RNA in blood	Pos^b^	Pos^b^	ND	ND	Pos^b^	USUV	ND	ND	USUV	ND
Flavivirus RNA in urine	ND	Neg	ND	ND	Neg	USUV	ND	ND	USUV	ND

CSF: cerebrospinal fluid; ELISA: enzyme-linked immunosorbent assay; IFA: immunofluorescence assay; NAT: nucleic acid testing; ND: test was not done; Neg: negative; NT: neutralising antibody titres; Pos: positive; USUV: Usutu virus.

^a^ Threshold cycles are reported between brackets. Values ≤ 40 are considered positive.

^b^ Pan-flavivirus nested RT-PCR was positive, but sequencing was unsuccessful.

### Case 1: Patient with encephalitis

Mid-July 2018, a male patient in his late 60s with an underlying malignancy, hypertension and diabetes mellitus was admitted to the Neurology Department of Padova City Hospital with acute onset of confusion. He had been complaining about a mild headache in the previous 3 days leading up to admission. His pulse rate, blood pressure and body temperature were normal. The neurological examination revealed language disturbances (aphasia), dressing and ideomotor apraxia and temporal disorientation. Routine blood tests showed an increase in the C-reactive protein level. A brain CT scan and MRI was unremarkable and excluded a vascular aetiology of symptoms. About 6 hours after these tests, his neurological status worsened and the patient developed severe confusion and apraxia. A lumbar puncture was performed and CSF showed a mild increase of protein level (0.82 g/L, norm: 0.15-0.45 g/L) and monocyte cell count (8-mononucleate leukocytes/µL with no erythrocyte). Suspected viral encephalitis was diagnosed and empirical antiviral therapy with acyclovir was initiated and continued for 10 days. Screening with the FilmArray Meningitis/Encephalitis panel (BioMerieux) excluded the presence of common pathogens that cause central nervous system infections (i.e. *Escherichia coli*, *Haemophilus influenzae*, *Listeria monocytogenes*, *Neisseria meningitidis*, *Streptococcus agalactiae*, *Streptococcus pneumoniae*, cytomegalovirus, enterovirus, human parechovirus, herpes simplex virus type 1, herpes simplex virus type 2, human herpesvirus 6, varicella-zoster virus, *Cryptococcus neoformans*, and *C. gattii*). The patient underwent screening to exclude autoimmune causes of encephalitis (onconeural and anti-neuronal surface antibodies).

As recommended by the surveillance plan for WNV and USUV infections for patients with meningitis/encephalitis, the patient was tested for WNV and USUV and USUV RNA was found in blood samples. During the following 72 hours symptoms progressively improved, aphasia and apraxia completely resolved and the patient was discharged at day 10 with a full recovery. A second MRI of the brain and electroencephalography performed at day 4 post-discharge were normal. Virological testing performed during follow-up showed the persistence of USUV RNA in blood up to 40 days after symptom onset i.e. the time of the last evaluation, notwithstanding clinical recovery (Table 2).

### Case 2: Patient with fever and arthralgia

End-August 2018, a male patient in his late 80s, with hypertension and ischaemic heart disease, was admitted for intermittent fever (temperature up to 39.2 °C), headache and arthralgia (especially in the lower limbs), which started the day before admission. Similar symptoms had occurred 3 weeks before admission and lasted for 3 days. At admission, the patient was in good physical condition and neurological examination was unremarkable. Blood routine tests only showed mild thrombocytopenia. Blood cultures were negative, while testing for arbovirus infection confirmed USUV infection. The patient, who received treatment with paracetamol and intravenous hydration, fully recovered in 6 days. This patient had persistence of USUV RNA in blood and urine during follow-up, with viral RNA still detectable in blood on day 34 after symptom onset (Table 2).

### Case 3: Patient with fever, headache and myalgia

Mid-August 2018, a male patient in his early 90s, with hypertension, ischemic heart disease and with underlying malignancy, went to the emergency ward for intermittent fever (temperature up to 38.5 °C), severe headache and myalgia. Blood testing showed increased C-reactive protein level and reduced lymphocyte and platelet counts. Empirical antibiotic therapy with amoxicillin/clavulanic acid was started. Because of the persistence of symptoms, the patient was hospitalised, for 10 days mid- to end-August 2018. His brain CT scan and neurological examination were unremarkable. Blood and urine cultures were negative and laboratory tests excluded common infectious causes of fever. During hospitalisation, the patient received treatment with ceftriaxone, paracetamol and hydration, with a resolution of fever in 3 days. Testing for WNV and USUV infection performed on day 15 after symptom onset demonstrated the presence of USUV RNA in blood and urine. Follow-up testing on day 23 after symptom onset showed the persistence of USUV RNA in blood and urine (Table 2).

### Case 4: Viraemic blood donor

Mid-August 2018, a healthy asymptomatic male in his late 50s donated blood, which was repeatedly reactive in WNV NAT screening. After 2 days, the blood donor developed arthralgia and myalgia lasting for 5 days. Routine blood and urine test results were unremarkable. Probably due to the low viral load, testing by WNV and USUV specific real-time RT-PCR assays gave negative results both at baseline and during follow-up. Serology testing demonstrated the presence of USUV IgM antibodies and the appearance of USUV IgG during follow-up, confirmed by neutralisation assay. On day 25 after the index donation, USUV and WNV neutralisation titres were 160 and 40, respectively.

### Cases 5–8: Patients with influenza-like symptoms

Between around mid- August and mid- September 2018, three male patients and one female patient (age range: 17–80 years) reported influenza-like symptoms. Symptoms included fever > 38.5 °C, headache, myalgia, asthenia and in two cases, rash. In all cases, complete recovery occurred within 2 weeks after symptom onset. These patients had high levels of USUV IgM antibodies in serum and titres of USUV-neutralising antibody that were fourfold higher than WNV-neutralising antibody titres and ranged between 160 and 1,280. Unfortunately, for these cases, blood and urine samples were not available for molecular testing. Since these patients had also positive WNV IgM and IgG ELISA and WNV-neutralisation test, we cannot exclude a recent WNV infection or even WNV and USUV co-infection.

### Virological findings in patients with USUV infection

The results of laboratory tests for the differential diagnosis between WNV and USUV infection are detailed in Tables 1 and 2.

**Figure 2:**
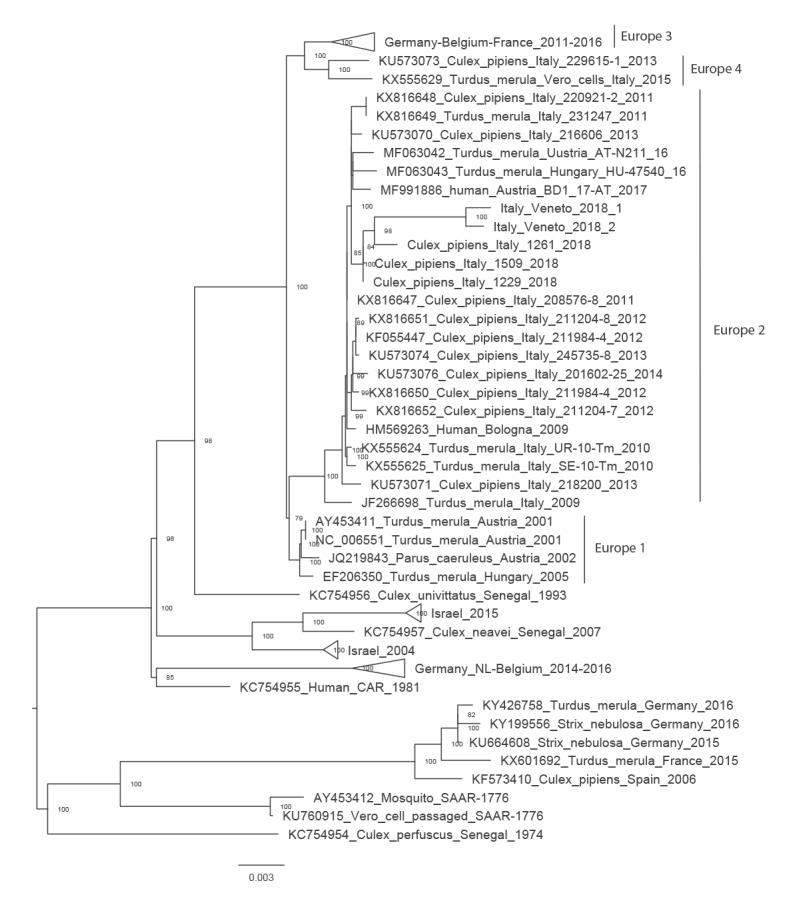
Phylogenetic analysis of Usutu virus sequences detected in clinical samples, northern Italy, 2018

NCBI: National Center for Biotechnology Information; USUV: Usutu virus.

In cases 1, 2 and 3, USUV RNA was detected by two different real-time RT-PCR methods on both whole blood and urine samples, while WNV real-time RT-PCR was negative (Table 2). In case 1 and 2, USUV RNA was tested also in CSF and saliva, respectively, with negative results. USUV infection was confirmed in case 2 and 3 also by pan-flavivirus nested RT-PCR and viral genome sequencing (GenBank accession numbers MK591822 and MK591823, respectively) (Figure 2). Attempts to isolate the virus in Vero cells from serum and urine samples were unsuccessful.

The partial sequences of USUV Italy/Veneto/2018/1 (GenBank accession MK591822) and Italy/Veneto/2018/2 (GenBank accession MK591823) detected in the whole blood of patients with USUV infection (cases 2 and 3, respectively) were analysed using a dataset of 154 full genome nt sequences (from START to STOP codons of the polyprotein). This dataset included the USUV sequences deposited in GenBank (NCBI) as of February 2019, cited in Calzolari et al. [31], and the USUV sequences from three pools of *Culex pipiens* (*Culex_pipiens*/Italy/1261/2018, *Culex_pipiens*/Italy/1509/2018, *Culex_pipiens*/Italy/1229/2018) collected in the Verona, Venice and Padua provinces, respectively, in 2018. Alignment was performed with a fast and progressive method (FFT-NS-2) implemented in MAFFT V.7.0 (https://mafft.cbrc.jp/alignment/server/) [32]. The phylogenetic tree was obtained using the maximum likelihood method implemented in IQ-TREE-1.6.7 [33], with 10,000 ultrafast bootstrap replicates and with best-fit model GTR + F + R2 (General time reversible model + Empirical base frequencies + the FreeRate model with two categories) automatically identified by the software. FigTree 1.4 is the graphical viewer used to visualise the obtained phylogenetic tree.

A remarkable finding of this study was the persistence of USUV RNA in the blood of three patients during follow-up (Table 2). In case 1, USUV-RNA levels of ca 10^3 genomic copies/mL were still detectable in whole blood at 40 days after the onset of symptoms, while USUV RNA was detectable in urine up to the eighth day after onset (ca 4 x 10^3 genomic copies/mL). In case 2, USUV-RNA was still detectable in whole blood on day 34 after onset (ca 2 x 10^5 genomic copies/mL) and in urine up to 28 days after onset (ca 4 x 10^4 genomic copies/mL). In case 3, USUV RNA was detectable in two whole blood samples and two urine samples collected on days 15 and 23 after symptom onset (USUV RNA load was ca 10^5 genomic copies/mL in all samples). No further blood and urine samples from these patients were available for testing because the patients had difficulty continuing follow-up.

Serum USUV IgM and IgG antibodies were detected in all patients by in-house developed IFA, but cross-reacting WNV IgM and IgG antibodies were also detected by both in-house IFA and ELISA assays (Table 1). Neutralisation assays demonstrated the presence of high titre USUV-neutralising antibodies in all patients and WNV neutralising antibodies (at lower titre than USUV-neutralising antibodies) in seven patients.

### Genome sequencing and phylogenetic analysis of USUV

Partial sequencing and phylogenetic analysis of USUV genome from cases 2 and 3 (Italy/Veneto/2018/1 and Italy/Veneto/2018/2, respectively) showed > 99.9% sequence identity between the two viruses and clustering within the Europe 2 lineage. In the phylogenetic tree, the two human USUV sequences had the highest nt similarity (99.1–99.4%) with USUV sequences obtained from three pools of *Culex pipiens* collected in the Veneto Region in 2018 (Figure 2).

## Discussion

The spectrum of clinical presentations of USUV infection in humans is largely unknown with only a few cases reported in the literature. Here, we describe the clinical and virological findings in eight cases of symptomatic USUV infection, one with encephalitis and seven with ILI, who were investigated at tertiary hospitals and at the regional reference laboratory of the Veneto Region.

USUV encephalitis occurred in a patient in his 60s, who had clinical conditions that represent risk factors for flavivirus neuroinvasive disease, i.e. hypertension, diabetes mellitus and an underlying malignancy. Co-morbidities were present also in most cases of USUV neuroinvasive disease that have been reported so far in the literature [6,16-18]. The patient developed a mild and self-limiting form of encephalitis with normal electroencephalography and no signs of disease in brain MRI; the diagnosis was supported by the acute onset of confusion, aphasia and apraxia with mild CSF pleocytosis [27].

So far, only two cases of USUV-related fever have been reported in the literature [15]. It is conceivable that the occurrence of USUV fever is underestimated, however, as its symptoms may be misdiagnosed as West Nile fever and because surveillance programs for USUV infection in humans generally target only neuroinvasive disease. Also, USUV infection poses the problem of the differential diagnosis with WNV infection due to the high genetic and antigenic similarities between the two viruses. In the cases reported here, IFA and ELISA serology assays could not discriminate between WNV and USUV infection; neutralisation assays showed higher titres of USUV-neutralising antibodies than WNV-neutralising antibodies, but could not exclude a recent WNV infection or even USUV/WNV coinfection. As expected, false-positive results were obtained with the WNV NAT used for donor screening, which is known to cross-react with USUV and other flaviviruses. These observations emphasise the need to develop more specific serology assays for the diagnosis of flavivirus infections and the relevance of using specific molecular assays for case confirmation.

In three patients, USUV RNA persisted in blood and urine after symptom onset, similar to what has been observed in patients with WNV infection [21,28,29]. In these patients, USUV RNA was still detectable in whole blood at the last follow-up visit 23–40 days after symptom onset. USUV RNA persisted also in urine, up to 28 days after symptom onset. Detection of USUV RNA in whole blood and urine a long time after symptom onset is a novel finding in Usutu patients and may confirm the usefulness of molecular testing in whole blood and urine for the diagnosis of acute flavivirus infection [21,29]. Viral RNA load in blood was relatively high, ranging from ca 10^3 genome equivalents/mL to 10^5 genome equivalents/mL. Similar viral load can be observed in patients with WNV infection [21,30].

An increase of USUV activity with human cases of infection have been reported in several European countries, including Italy [13,14]. Sequencing of the USUV genome from both human cases and mosquitoes collected from pools in the Veneto Region showed that the virus clustered with the currently most widespread Europe 2 lineage [13,14]. In the Veneto Region, USUV-positive mosquitoes were widely distributed in the territory, while most human cases of USUV infection were resident in a relatively small geographical area. This difference could be due to biases in the intensity of surveillance in humans, but we cannot exclude the emergence of a strain with higher pathogenic potential in the area.

### Conclusion

The results of this study showed a significant overlap between USUV and WNV infection that challenged differential diagnosis. Symptoms associated with USUV infection included encephalitis in one case and ILI in seven cases. Causality between USUV infection and disease could not be proven in this study and it cannot be excluded that USUV infection was an incidental finding in subjects with other diseases. Thus, further studies are warranted to clarify the role of USUV in human disease. While waiting to understand the clinical relevance of USUV, surveillance programs for WNV and USUV should be strengthened in relevant areas.
